# Cell lipid biology in infections: an overview

**DOI:** 10.3389/fcimb.2023.1148383

**Published:** 2023-10-06

**Authors:** Luan Gavião Prado, Niels Olsen Saraiva Camara, Angela Silva Barbosa

**Affiliations:** ^1^ Laboratório de Bacteriologia, Instituto Butantan, São Paulo, Brazil; ^2^ Departamento de Microbiologia, Instituto de Ciências Biomédicas, Universidade de São Paulo, São Paulo, Brazil; ^3^ Laboratório de Imunobiologia de Transplantes, Departamento de Imunologia, Instituto de Ciências Biomédicas, Universidade de São Paulo, São Paulo, Brazil; ^4^ Disciplina de Nefrologia, Departamento de Medicina, Universidade Federal de São Paulo, São Paulo, Brazil

**Keywords:** lipid, lipid metabolism, phospholipids, lipid droplets, bacterial and viral infections

## Abstract

Lipids are a big family of molecules with a vast number of functions in the cell membranes, within the cytoplasm, and extracellularly. Lipid droplets (LDs) are the most common storage organelles and are present in almost every tissue type in the body. They also have structural functions serving as building blocks of cellular membranes and may be precursors of other molecules such as hormones, and lipoproteins, and as messengers in signal transduction. Fatty acids (FAs), such as sterol esters and triacylglycerols, are stored in LDs and are used in β-oxidation as fuel for tricarboxylic acid cycle (TCA) and adenosine triphosphate (ATP) generation. FA uptake and entrance in the cytoplasm are mediated by membrane receptors. After a cytoplasmic round of α- and β-oxidation, FAs are guided into the mitochondrial matrix by the L-carnitine shuttle system, where they are fully metabolized, and enter the TCA cycle. Pathogen infections may lead to impaired lipid metabolism, usage of membrane phospholipids, and LD accumulation in the cytoplasm of infected cells. Otherwise, bacterial pathogens may use lipid metabolism as a carbon source, thus altering the reactions and leading to cellular and organelles malfunctioning. This review aims to describe cellular lipid metabolism and alterations that occur upon infections.

## 1 Introduction

Lipids are usually used as energy-storage depots, structural building blocks, and as second messengers in signaling cascades. They accumulate in lipid-storage specialized tissues, such as the adipose one, but are present in almost every cell type due to their importance as energy and carbon skeleton sources, and as hormone precursors. Through β-oxidation, a process in which a series of enzymes oxidize long, medium, or short FAs, cells yield much more ATP than using glucose or glycogen as primary sources. High energy-demanding cells, such as tubular epithelial cells, colonocytes, and myocytes, often use this energy pathway as their main metabolic pathway. Perturbation on lipid metabolism and its enzymes, as well as dietary overload and obesity, lead to cytoplasmic lipid accumulation and, consequently, lipotoxicity (Kang et al., 2015; Adeva-Andany et al., 2019; Cockcroft, 2021).

In general, lipids are a very diverse group of molecules, but with one characteristic in common: insolubility in water. Storage lipids are often fats, and their derivatives are FAs. They are composed of hydrocarbons, with a carbon skeleton, and may be branched or not. Unsaturation is characterized by a double bond between two carbon atoms, in a *cis* or *trans* configuration. Oxidation of FAs is a very exergonic reaction, thus producing a lot of energy that is converted into ATP in the mitochondrion (Cockcroft, 2021).

FAs uptake is mediated by three main transporters, CD36 or fatty acid translocase (FAT), fatty acid binding protein (FABP), and fatty acid transport protein (FATP). After the entrance into the cell cytoplasm, very long- and long-chain FAs are initially α- and β-oxidized in peroxisomes, and their metabolites, together with medium-chain FAs enter mitochondria through the L-carnitine shuttle system to start a new round of β-oxidation. At the end, acetyl-CoA is generated and enters the TCA cycle (Mannaerts et al., 2000; Luiken et al., 2002; Jansen and Wanders, 2006; Storch and McDermott, 2009; Schwenk et al., 2010; Anderson and Stahl, 2013; Melton et al., 2013).

Phospholipids are the major components of biological membranes and are composed of two hydrophobic fatty acyl chains and one hydrophilic head group. The fatty acyl chains are responsible for biological and physical properties affecting all biological processes that occur in the inner and outer membrane leaflets. Alterations in the fatty acyl chains are orchestrated by reacylation and deacylation, known as the Land’s cycle (Van Meer et al., 2008; Wang and Tontonoz, 2019; Cockcroft, 2021). Perturbance of phospholipid content in the cell membrane may be involved in the pathogenesis of some diseases, including bacterial infections.

Intracellular pathogens have well-defined mechanisms to exploit the host’s cell lipid metabolism and use those molecules as an energy source for their metabolic processes. In extracellular pathogens, lipid metabolism impairment and metabolism exploitation are still not completely elucidated (Walpole et al., 2018; Libbing et al., 2019).

Besides lipids’ roles in disease pathogenesis, there is a crescent body of information outlining lipid droplets (LDs) as a hub that integrates metabolic and inflammatory responses to face infections (Monson et al., 2021b). In viral infections, LDs are used as the site of viral replication, since it is partially protected against the machinery of pathogen killing. They also use the lipid content from LDs to build up their capsids (Samsa et al., 2009; van der Meer-Janssen et al., 2010; Farías et al., 2022). Furthermore, foamy macrophages, a hallmark of tuberculous lesions, are generated after *Mycobacterium* species induction of LDs formation (Agarwal et al., 2021).

This review aims to describe lipid metabolism and exploitation of lipids, lipid metabolism, and cell membrane phospholipids by pathogens, as well as their consequences to cells and organisms.

## 2 Lipid metabolism

Lipids are molecules composed of a hydrocarbon chain, branched or not, with a carboxyl-terminal group. As mentioned above, unsaturated FAs have a double bond between two carbons in the *cis* or *trans* position. Saturated FAs have linear chains with no double bonds along them, that may be as small as those with two carbons (acetic acid) or as large as those with 26 carbons (cerotate) (Adeva-Andany et al., 2019).

Lipid metabolism is a very complex process that involves both anabolic and catabolic reactions. The process starts with FA degradation and absorption by the gastrointestinal tract and the interaction of lipids with the gut microbiome. As this particular subject is beyond the scope of this review, the interested reader may search for additional information, well provided by Schoeler and Caesar (2019), who extensively discuss this aspect of lipid metabolism. Here we will focus on intracellular lipid metabolism and its importance for maintenance of the cellular functioning.

CD36, also known as fatty acid translocase, is a receptor of the scavenger receptor family that mediates the uptake of FAs from the extra- to the intracellular milieu (Luiken et al., 2002; Pepino et al., 2014; Hou et al., 2015; Hua et al., 2015; Yang et al., 2017; Jay and Hamilton, 2018). It is an integral membrane protein, highly glycosylated, and present in erythrocytes, platelets, immune cells, adipocytes, enterocytes, and epithelial cells (Pepino et al., 2014). Although it has a well-described function as a FA transporter, CD36 is also involved in the recognition of other molecules, such as thrombospondin, collagen, *Plasmodium*-infected erythrocytes, and oxidized-LDL (Nicholson et al., 2001; Silverstein and Febbraio, 2010; Jay and Hamilton, 2018; Maréchal et al., 2018; Pombinho et al., 2018).

It is known that CD36 production is augmented during acute and chronic kidney diseases, which is associated with epithelial-to-mesenchymal transition (EMT) and inflammation in animal and cellular models of kidney injury (Hou et al., 2015; Hua et al., 2015; Yang et al., 2017). Endothelial cells are also prone to undergo endothelial-to-mesenchymal transition after infection or disturbance of cell functioning. In primary endothelial cells infected by *Leptospira interrogans* CD36 is overexpressed in the cell membrane, as evidenced by immunofluorescence microscopy (Sato and Coburn, 2017).

Treatment of bovine mammary epithelial cells with palmitate leads to CD36 gene overexpression, in a dose-dependent manner. Cells treated with as much as 600 µM of palmitate have CD36 gene expression augmented at almost 40-fold compared to those without any treatment, which highlights the importance of this receptor in lipid uptake, and how lipid intake may influence its concentration in the cell membrane (Qi et al., 2014).

Other receptors are involved in lipid uptake to the cytoplasm, such as fatty acid transporter proteins 1 to 6 (FATP 1-6) and fatty acids binding proteins (FABPs) (Melton et al., 2013). FATPs are also integral membranes with different roles, according to each type. Collectively they are described as membrane-bound proteins that act in the uptake of long-chain FAs and in the activation of FAs, as the first step of β-oxidation, i.e., acylation, of very long-chain FAs (DiRusso et al., 2005; Anderson and Stahl, 2013; Melton et al., 2013).

FABPs have a high affinity to long-chain FAs and bind both saturated and unsaturated FAs (Storch and McDermott, 2009). There are multiple FABPs functioning similarly, and the affinity to a given FA increases as hydrophobicity rises. There are 9 different FABP types described to date, known as FABP 1-9 (Ono, 2005; Li et al., 2020). Different isoforms are named after the main organ where they were described (Furuhashi, 2019). These proteins, also characterized as chaperones, exert functions in FA transport and in intracellular communication (Gorbenko et al., 2010).

Besides being internalized by receptors, endocytosis is also used as a means of transporting lipids into the cell cytoplasm. The most well-studied forms of general endocytosis are the ones mediated by clathrin and caveolin (Johannes et al., 2016; Kaksonen and Roux, 2018; Kostarnoy et al., 2022; Abdulova et al., 2023; Liu et al., 2023; Tang et al., 2023; Yu and Yoshimura, 2023). More recently, a new mechanism of lipid transport and uptake, named Mincle-mediated endocytosis (MiMe) was described. The Mincle receptor was first reported as a pattern recognition receptor (PRR) and plays a role in triggering inflammatory responses after the recognition of fungal and bacterial lipids. However, it also mediates lipid uptake, as the knockout of Mincle leads to a pronounced decrease in lipid internalization by brain vessel endothelial cells.

Free FAs, or albumin-bound FAs are present in the bloodstream and enter the cells by their transporters, as described above. After their entry into the cytoplasm, FAs are activated by the enzyme fatty acyl CoA synthetase that catalyzes the binding of an Acyl-CoA to each acetyl moiety. Very long-chain FAs must be shortened by peroxisomal α-oxidation before entering mitochondria (Adeva-Andany et al., 2019).

Carnitine palmitoyl transferase 1 (CPT1) then catalyzes the transfer of acylcarnitine to the inner mitochondrial membrane. This process occurs with the acyl groups being transferred from Coenzyme A (CoA) to L-carnitine, generating the acylcarnitine esters that are allowed to pass through the mitochondrial outer membrane. Carnitine acyl-carnitine translocase (CACT) transports the acyl-carnitine esters into the mitochondrial matrix in exchange for a free carnitine. Finally, Carnitine palmitoyl transferase 2 (CPT2) catalyzes fatty acyl-CoA esters import into the mitochondria, transferring the acyl groups from the acyl-carnitine esters, generating acyl-CoA esters (Adeva-Andany et al., 2019).

In the mitochondrial matrix, Acyl-CoA is cleaved from FA-Acyl-CoA by β-oxidation and enters the TCA cycle to generate ATP (Berndt et al., 2019; Gewin, 2021). The whole β-oxidation process is catalyzed by only four different enzymes, e.g., Acyl-CoA dehydrogenase, Enoyl-CoA hydratase, 3-L-hydroxyacyl-CoA dehydrogenase, and β-ketothiolase. After the four rounds, there is generation of an acetyl-CoA that enters the TCA pathway, and an Acyl-CoA ester, with two carbons less. These iterative rounds keep happening until total Acyl-CoA esters are consumed (Adeva-Andany et al., 2019).

Conversely, acetyl-CoAs that are produced in the mitochondria and are not used in TCA are exported to the cytosol, and back to the ER, where they are used in lipogenesis, contributing to the biosynthesis of neutral lipids and phospholipids (Wältermann et al., 2007; Turchetto-Zolet et al., 2011; Hsu et al., 2023) *De novo* lipid synthesis or lipid biogenesis is governed by the mTORC1 signaling pathway (Düvel et al., 2010; Li et al., 2010; Peterson et al., 2011; Owen et al., 2012; Han et al., 2015; Lee et al., 2017), which is responsible for lipogenic enzyme production by SREBP 1 and 2 induction or reduction of their inhibitors (Düvel et al., 2010; Li et al., 2010; Peterson et al., 2011; Owen et al., 2012; Han et al., 2015). Another important role of the mTORC pathway is mediated by SRKP2 that induces more efficient mRNA splicing of lipogenic enzymes, thus enhancing *de novo* lipid synthesis (Lee et al., 2017).

In this way, lipids are produced after the generation of Acetyl-CoA, in the TCA cycle, from either citrate or acetate by ATP citrate lyase (ACLY) or acyl-CoA synthetase short-chain (ACSS). Fatty acid synthesis then takes place by using Acetyl-CoA and Malonyl-CoA, catalyzed by Fatty Acid Synthetase. The addition of more acetyl-CoA will lead to the formation of a palmitate molecule which is then used to generate longer FAs by elongation processes, unsaturated FAs catalyzed by stearoyl-CoA desnaturase 1 (SCD1), and finally phospholipids and signaling lipids (Lee et al., 2017). Although produced in the mitochondria, the cytosolic acetyl-CoA pool is used by lipogenic enzymes for FA biosynthesis. *In vitro*, cells under glucose restriction use more acetyl-L-carnitine to generate acetyl-CoA and consequently, fatty acids. Therefore, L-carnitine not only participates in the L-carnitine shuttle but also represents a carbon source to cells under physiological or hypoglycemic environment (Hsu et al., 2023).

Other important mechanism involved in cell homeostasis and pathogen response is macroautophagy, or simply autophagy. During this process, cells engulf and destroy organelles to maintain proper functioning. The phagophore, a double-membrane vacuole, is responsible for engulfing the organelles and fusing them with lysosomes where hydrolysis occurs. The formation of the phagophore is mediated by membrane donation from healthy organelles, but also by *de novo* synthesis of FAs in the ER, catalyzed by long-chain Acyl-CoA synthetase (ACS), in particular by the enzyme Faa1 (Schütter et al., 2020).

Triacylglycerol, a glycerolipid, is used as storage and is composed of three FAs attached to the three hydroxyl groups of the glycerol backbone (Cockcroft, 2021). Cell membranes are composed of three main lipids: glycerophospholipids, sphingolipids, and sterols, such as cholesterol. Mammalian cells synthesize and yield phospholipids that are not present in prokaryotes, such as phosphatidylserine, phosphatidylcholine, phosphatidylinositol, phosphatidylethanolamine, and phosphatidylglycerol. The percentage of each molecule varies according to the cell type and environment (Wang and Tontonoz, 2019; Cockcroft, 2021).

Neutral lipids are synthesized in the ER mainly as triacylglycerol and are stored in LDs. The most important enzymes, that are rate limiting in the synthesis of triacylglycerol, are acyl-CoA diacylglycerol acyltransferase (DGAT) 1 and 2, responsible for catalyzing the acylation of sn-1,2-diacylglycerol, using acetyl-CoA as a substrate. Evolutionary studies have shown that these enzymes are conserved in all eukaryotes, despite molecular and genetic differences (Turchetto-Zolet et al., 2011). Inhibition of DGAT 1 and 2 leads to decreased LD formation, and to impaired viral replication and bacterial survival during infections (Monson et al., 2021a; Yuan et al., 2021; Kiarely Souza et al., 2022; Dias et al., 2023).

Membrane phospholipid synthesis occurs in the ER. Phosphatidic acid (PA) is used as the precursor of most of the other phospholipids in the host’s cells. A two-round acylation of glycerol-3-phosphate is mediated by the enzymes Glycerol-3-Phosphate Acyltransferase (GPAT) and Lyso-PA Acyltransferase (LPAAT), with PA as an end product. From PA, other phospholipids, such as phosphatidylcholine, phosphatidylethanolamine, and phosphatidylserine are produced. Phosphatidylglycerol and cardiolipin are synthesized in the mitochondria. After their synthesis, phospholipids are addressed to the cytoplasmic and other membranes in the cell and are integrated into them (Van Meer et al., 2008; Cockcroft, 2021).

## 3 Lipid droplets and their roles in physiology and pathology

LDs are important organelles for cellular functioning and are constituted by high hydrophobic neutral FAs, within an aqueous environment. LDs are present in almost every cell in the organism and are responsible for FAs and specific protein storage, besides taking part in other complex processes, such as viral replication (Walther and Farese, 2012).

A family of proteins named perilipin (PLINs) is involved in lipid uptake, coating, and addressing LDs inside cells (Frank et al., 2015; Najt et al., 2022). Perilipin 1 was the first perilipin described (Greenberg et al., 1991) and after its discovery, the study of LDs, their lipidomic and proteomics, and the role of LDs in health and disease have increased dramatically, expanding the LD biology field (Kobayashi et al., 2008; Kuerschner et al., 2008; García-Arcos et al., 2010; Turchetto-Zolet et al., 2011; Hsieh et al., 2012; Lin et al., 2014; Markgraf et al., 2014; Kamili et al., 2015; Li et al., 2015; Viktorova et al., 2018; Freyre et al., 2019; Guyard et al., 2022; Kim et al., 2022; Oluwadare et al., 2022).

Assembly of LDs in the cytoplasm is thought to occur in very close proximity to the ER, but the exact mechanism by which those organelles are formed is still not completely understood. It has been shown that ER, mitochondria, and LDs are in close contact during LD formation and that this contact is mediated by two proteins called oxysterol binding protein (OSBP)-related protein (ORP) 5 and ORP8 (Guyard et al., 2022). Besides those, PLIN2 is involved in FA uptake and in addressing it in the forming LD (Hsieh et al., 2012; Najt et al., 2022). Other protein that may be involved in the inflammatory response and LD formation is the cell death-inducing DFF45-like effector b (Cideb). Mice lacking this protein are more prone to developing severe DSS-induced colitis and even more severe DSS-induced colitis if they undergo a high-fat diet (Sun et al., 2017). After the formation of a LD, enlargement is achieved by the fusion of multiple droplets. Besides the phospholipid monolayer, that separates LDs from the aqueous environment of the cytoplasm, there are also specific proteins attached to or in close relation with the LD’s phospholipid monolayer (Walther and Farese, 2012).

Usage (lipolysis) or storage (lipogenesis) of lipid content from LDs are very strictly regulated. Insulin and glucagon participate in this regulation, as they are major regulators of metabolism (Walther and Farese, 2012). Within the cell, the mechanism of new LD formation is still under debate, but the production of triacylglycerols and sterol ester by enzymes localized in the ER and the budding of the nascent LD from the ER membrane is one of the proposed mechanisms. Otherwise, usage of lipid content from LDs is mediated by lipases, such as the Adipose Triacylglycerol Lipase (ATGL) that converts triacylglycerol to diglyceride, and the Hormone-sensitive Lipase (HSL) that breaks diglyceride into monoglyceride (Walther and Farese, 2012; Monson et al., 2021b). The involvement of other cellular mechanisms in lipid metabolism has been described, though yet not fully understood (Singh et al., 2009; Yan et al., 2018; Jarc and Petan, 2019).

Autophagy seems to control LD digestion and lipolysis. Inhibition of autophagy in hepatocytes by knocking down the *Atg5* gene leads to an augmentation of the number and size of LDs when cells are treated with oleate overload compared to cells in resting conditions. Lipid accumulation in those cells is probably mediated by autophagy inhibition, leading to impaired lipolysis (Singh et al., 2009).

Interferons play a specific role in the generation of LDs, especially during viral infection. Interferon-stimulated genes (ISG) encode some important proteins that act as inhibitors of LD formation (Helbig et al., 2013; Peña Cárcamo et al., 2018; Bosch et al., 2020; An et al., 2022). One of those proteins is the Sterile Alpha Motif and Histidine-aspartate Domain containing protein 1 (SAMHD1). In an HCV infection model, cells that overexpress this protein had 50% less LDs compared to the control and, consequently, produced less virus. On the contrary, the knockdown of the SAMHD1 led to an increase in HCV core protein detection (An et al., 2022). Other ISG that influences LD formation is Viperin. In homeostatic conditions this protein is localized in the ER and is transported to LDs. Viperin also exerts an antiviral role, during infection by Junín mammarenovirus (Peña Cárcamo et al., 2018).

LDs are also the site of eicosanoid production since they are a reservoir of arachidonic acid (Moreira et al., 2009). In Caco2 cells, the presence of cyclooxygenase-2 (COX-2) and prostaglandin E_2_ (PGE) was described and correlated to inflammation during colon cancer (Accioly et al., 2008). In oleic acid-treated IEC-6 cells, the formation of new LDs overlaps the compartmentalization of cytosolic PLA_2_, with production and storage of PGE_2_ within the LDs (Moreira et al., 2009). In the HL-60-derived neutrophils, treatment with *Porphyromonas gengivalis* led to an increased number of LDs, with a concomitant increase in Perilipin 3 protein. Interestingly, there is also an increase in PGE_2_ production and release, which demonstrates that there is a relation between the production of LDs and its proteins, and the production and release of eicosanoids (Nose et al., 2013).

### 3.1 The role of LDs in viral infection

Viral infection is known to induce LD accumulation in the cell cytoplasm. Both Dengue and Zika viruses were shown to induce LD accumulation in vertebrate and invertebrate hosts. Hepatitis C, Herpes simplex-1, Influenza-A, and SARS-CoV-2 also induce LDs in human and mouse models. Viruses use LDs to evade the host´s innate immune system to replicate and form new viral particles (Barba et al., 1997; Samsa et al., 2009; Barletta et al., 2016; Episcopio et al., 2019; da Silva Gomes Dias et al., 2020; Monson et al., 2021a).

SARS-CoV-2, the agent of COVID-19, uses cells’ machinery to induce the production of triacylglycerol and, consequently, LD formation. Those are used by the virus to guarantee a high rate of virion replication. The virus nucleocapsid protein activates DGAT1 and 2 expression, and adipocyte differentiation-related protein (ADRP) is also overexpressed, leading to the formation of more LDs. DGAT inhibition by silencing RNA or pharmacologically, by xanthohumol, leads to lower yields of virus *in vitro* and a better response to infection *in vivo*, respectively (da Silva Gomes Dias et al., 2020; Yuan et al., 2021). Another viral protein that is involved in the accumulation of LDs during COVID-19 is ORF3a. The protein itself is capable of inducing LD accumulation by impeding autophagy flux in the cells. Mutations in the ORF3a protein, such as T223I and S171L abrogated induction of LDs and led to deficient replication and lower virus yield *in vitro* (Wang et al., 2023).

Flaviviruses, such as Zika virus and Dengue virus, are widely known to use cellular lipids to replicate and induce the host’s inflammatory response. Zika virus induces LD formation during the early stage of infection and depletes LD storage during late-stage infection. Inhibition of autophagy in Zika virus-infected MDCK cells leads to LD content reduction and, consequently, viral replication reduction. There is also evidence that DGAT-1 blockage in neural cells and in mice reduces viral replication by blocking LD formation in those cells (Ambroggio et al., 2021; Monson et al., 2021a; Dias et al., 2023; Stoyanova et al., 2023).

As mentioned above, viral infection leads to transient LD induction, and type I- and III-interferon (IFN) mRNA levels are also elevated during the early stages of infection. Both LD and IFN levels increase as early as 2 hours post-infection for LDs and 8 hours post-infection for IFN, remain elevated during the next 48 hours and return to baseline at 72 hours post-infection (Monson et al., 2021a).

### 3.2 Lipids and bacterial infection

Intracellular bacteria developed a wide range of mechanisms that allow them to invade and multiply inside the host’s cells, such as specialized phagocytes, or other cell types including epithelial or adipose cells. Those mechanisms include avoidance of lysosome fusion to phagosomes, and the ability of those pathogens to survive and grow inside phagocytic vacuoles. After entry into cells, those pathogens rely only on the host’s metabolism to obtain the energy necessary for survival and growth. Subversion of cell lipid metabolism, and the ability of the pathogen to use lipids from the hosts as building blocks and energy sources is one of the mechanisms that allow intracellular persistence (Gago et al., 2018; Walenna et al., 2018; Walpole et al., 2018; Libbing et al., 2019).

By exploiting the role of FABPs in controlling fat metabolism to obtain nutrients from host cells, intracellular microorganisms, such as *Chlamydia pneumoniae*, hijack FABP4 and induce fatty acid oxidation (FAO) to obtain ATP generated by the host cells, consequently reducing the number and size of LDs inside adipocytes and releasing high amounts of glycerol and FAs. Thus, the use of cellular FAO to fuel bacterial TCA allows replication, growth, and persistence inside the host’s cells (Walenna et al., 2018).

*Salmonella Typhimurium*, a facultative intracellular bacterium, induces LD biogenesis as early as 1-hour post-infection in macrophages. Differently, *Escherichia coli* and dead *S. Typhimurium* are not able to cause LD accumulation. This capacity to induce early LD accumulation is dependent on the type III secretion system and contributes to intracellular bacterial survival and proinflammatory response (Kiarely Souza et al., 2022).

The foamy macrophage is a hallmark of tuberculous lesions. Those are macrophages with extreme LD accumulation. Different from what was first described, IFN-γ induces LD formation in macrophages during *Mycobacterium tuberculosis* infection as a host response to infection, and, surprisingly, *M. tuberculosis* is not able to acquire lipids from IFN-γ induced LDs. Thus, they are not a lipid source for the bacteria inside macrophages (Knight et al., 2018). Although the use of lipids from the host’s LD may not seem indispensable for *M. tuberculosis* growth, *in vitro* infection of macrophages by the vaccine strain bacillus Calmette-Guérin induces LD formation by TLR2 and leads to PGE_2_ production and the presence of cyclooxygenase-2 inside the LDs (D’avila et al., 2006; Almeida et al., 2014). Given the participation of macrophages in the response to infection and the ability of *M. tuberculosis* to trigger the formation of LDs inside those cells, it has become clearer that LDs are not only used by pathogens as a carbon source but are also an innate immune response to infection (D’avila et al., 2006; Almeida et al., 2014; Kumar et al., 2020; Kiarely Souza et al., 2022).

**Figure 1:**
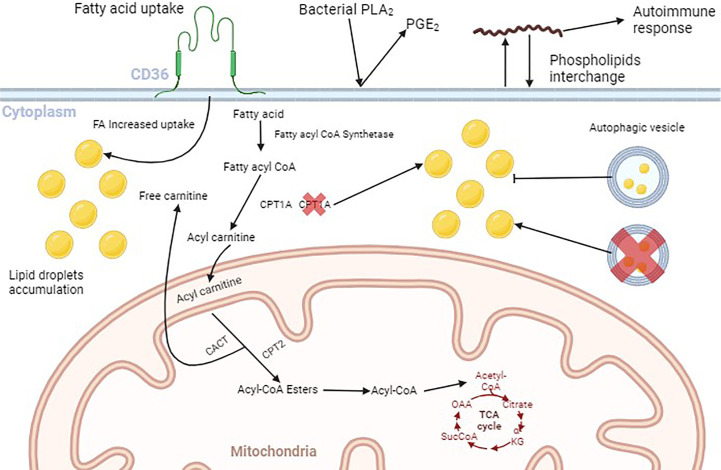
Schematic illustration of lipid fate within the cell. Fatty acids (FAs) bind to their membrane receptors and are translocated to the cytoplasm mediated by the FAs transporters (CD36, FABP, and FATP), where they are chemically modified. First, fatty acyl CoA synthetase adds an acyl CoA moiety to the FA. Then, Carnitine Palmitoyl Transferase 1A (CPT1A) converts the Fatty acyl CoA to Acyl carnitine and facilitates its passage through the mitochondrial outer membrane. Acyl carnitine enters the mitochondrial matrix in exchange for one free carnitine, mediated by Carnitine Acyl Carnitine Transferase (CACT). Carnitine Palmitoyl Transferase 2 (CPTA2) converts acylcarnitine to Acyl-CoA esters that are oxidized to Acyl-CoA that enters the Tricarboxylic Acid Cycle. The increase in the amount of membrane fatty acids receptors leads to the accumulation of cytoplasmic FAs, that are stored as lipid droplets (LDs). Inhibition of the autophagic flux is also responsible for LDs cytoplasmic accumulation. Extracellular pathogens such as *P. aeruginosa* produce enzymes like Phospholipase A_2_ (PLA_2_) that convert membrane phospholipids into Prostaglandin E_2_ (PGE_2_). Also, the spirochete *B*. *burgdorferi* can incorporate membrane phospholipids into their own membrane and then, reincorporate them into the cell membrane, leading to the production of antibodies underlying autoimmune responses. Figure generated by BioRender.com.

Since extracellular bacteria do not invade cells during infection, they evolved to exploit and use cells’ lipid storages from different locations, but also from LDs (Plotkowski et al., 2008). A brief explanation of FA transport to mitochondria, and how pathogens may interfere with lipid metabolism and cell membrane phospholipids is depicted in **Figure 1**.

Extracellular bacteria do not invade cells to escape immune responses or to obtain carbon and energy sources. However, they can exploit lipids from cell membranes. There is a growing body of evidence suggesting that extracellular pathogens are able to use host’s membrane phospholipids as a source of FAs to assemble their cell membrane, and also degrade phospholipids leading to the production of proinflammatory cytokines (Libbing et al., 2019). Also, *Helicobacter pylori* can glycosylate and use the host’s cholesterol to grow and evade the immune system (Wunder et al., 2006). Although these data point to the ability of some bacteria to use hosts’ lipids, the direct use of LDs as well as their induction or inhibition, and their role in direct cell lesions are still being investigated (Wunder et al., 2006; Plotkowski et al., 2008; Dulberger et al., 2020).

*Pseudomonas aeruginosa* produces a phospholipase-like protease, ExoU, an effector of the type III secretion system that induces the production of eicosanoid-mediated proinflammatory cytokines in an endothelial cell line and in a murine model of infection (Saliba et al., 2005). The presence of ExoU in *P. aeruginosa* isolates is related to antimicrobial resistance and the occurrence of tissue necrosis (Sharma et al., 2014; Subedi et al., 2018; Springer et al., 2019; Bagayoko et al., 2021; Deruelle et al., 2021). Although related to bacterial virulence, the protease needs a host chaperone to bind to the cell membrane and to induce cell rupture and inflammation. The DNAJC5 chaperone may be one of the chaperones involved in the process since human DNAJC5-deficient cells and *Drosophila* flies with the DNAJC5 orthologue knocked down are resistant to the necrosis effects caused by ExoU protease (Deruelle et al., 2021). *In vitro* studies showed that ExoU may interact with phosphatidylinositol-containing lipids and cleave them. Since cell membranes are composed of phosphatidylinositol-containing lipids, their composition may target it to ExoU action, leading to cell membrane rupture and tissue necrosis (Springer et al., 2019; Deruelle et al., 2021).

Another important enzyme produced by *P. aeruginosa* and secreted by its type V secretion system is PlpD, which displays a phospholipase A_1_ activity and has a catalytic domain that is quite similar to ExoU. PlpD is also able to bind mainly to phosphatidylinositol, but also to phosphatidic acid, phosphatidylserine, and phosphatidylglycerol (Da Mata Madeira et al., 2016). Despite the well-described binding of PlpD to phospholipids, the exact role of this enzyme in phospholipids’ cleavage is not completely understood. In contrast, a direct activity against phospholipids has been described for a PlpD homologue from *Fusobacterium nucleatum* (Trunk et al., 2019).

*Helicobacter pylori* infection is associated with gastritis, gastric ulcer, and gastric cancer. As the pathogen does not have any machinery to produce cholesterol, it relies only on the host’s cholesterol which is incorporated onto the bacterial cell membrane. *H. pylori* can glycosylate host cholesterol before incorporating it, and differences in glycosylation are associated with immune escape. By α-glycosylating cholesterol with the consequent generation of cholesteryl-α-glucosides they maintain themselves attached to the gastric mucosa and subvert neutrophils and macrophages action, delaying phagocytosis (Wunder et al., 2006; Du et al., 2016; Hsu et al., 2021; Qaria et al., 2021).

It has been postulated that *H. pylori* also excretes a vacuolating toxin, named VacA, which targets lipid rafts and sphingomyelin (SM), and is part of a whole mechanism that allows the bacterium to survive in the epithelium surface and extract nutrients from host cells, especially lipids. The association of multimeric VacA is dependent on low pH and the complex binds to the membrane as 26 nm oligomers. In supported lipid bilayers *in vitro*, VacA binds to SM plus cholesterol lipid bilayers without a clear lipid domain preference, but it binds to raft-like domains in lipid bilayers made of dioleoylphosphatidylcholine, SM, and cholesterol. Although it targets the above-mentioned lipids, there is a clear preference of VacA for phosphatidylserine in supported lipid bilayers, given its avidly for this phophplipid (Pagliaccia et al., 2000; Geisse et al., 2004; Gupta et al., 2008; Sewald et al., 2008; Gupta et al., 2010; Kim and Blanke, 2012; Raghunathan et al., 2018).

The use of host’s cell membrane lipids by extracellular pathogens is also considered a virulence factor. *Borrelia burgdorferi*, the causative agent of Lyme disease, exchanges lipids from lipid rafts with HeLa cells, *in vitro*. After getting cholesterol from the cell membrane, those spirochetes metabolize and integrate it into their cell wall. On the other hand, as they are capable of transferring metabolized cholesterol from their cell wall to the cell membrane, they target the cell to immune system attack (Östberg et al., 2007; Crowley et al., 2013).

The production of IgG anti-mammalian membrane phospholipids has been proposed as a diagnostic tool to follow the efficiency of Lyme disease treatment. Mice produce anti-phosphatidylglycerol, phosphatidylcholine, phosphatidic acid, phosphatidylserine, and phosphatidylethanolamine after infection by *Borrelia burgdorferi* cultured in a medium that contains those phospholipids. In human sera, the same pattern of IgG production against phospholipids has been described. In brief, these spirochetes are capable of incorporating host phospholipids, and this mechanism is probably related to the occurrence of symptoms related to autoimmunity, thus important to the pathogenesis of the disease (Arora et al., 2022; Gwynne et al., 2022).

Periodontal pathogens, *Porphyromonas gingivalis* and *Fusobacterium nucleatum* are phagocytosed and induce the formation of LDs within murine and human macrophage cell lines, i.e., Raw264.7 and THP-1. The proposed mechanism by which those pathogens induce LD accumulation is by disruption of lipid homeostasis and modulation of proteins and enzymes related to cholesterol influx and efflux, but not with the increase of cholesterol esterification. Lipid receptor expression, such as CD36 and FABP4, are also increased during macrophage infection by both pathogens, which can explain the augmented intake of free FAs or oxidized-LDL. These alterations in lipid metabolism are closely related to the formation of foamy macrophages and the occurrence of atherosclerosis and cardiovascular accidents (Kim et al., 2019; Rho et al., 2021).

Increased expression of the FA transporter CD36 is also observed in primary endothelial cell lines after infection by pathogenic leptospires, *L. interrogans* (Sato and Coburn, 2017). Interestingly, hamsters infected by pathogenic leptospires have circulating foamy macrophages (Putz et al., 2021). The occurrence of foamy macrophages is well described in mycobacterial infection, due to the accumulation of LDs in the cytoplasm of those phagocytes (Agarwal et al., 2021; Laval et al., 2021). We can speculate that this increase may lead to augmented uptake of FAs by the cells, and consequently accumulation of neutral lipids in the cytoplasm. However, the mechanism by which bacteria enhance CD36 and other surface lipid receptor expression is still not completely understood.

The interaction of pathogens with surface receptors and lipid rafts is necessary for adhesion, but also facilitates entrance into the cell, in the case of intracellular pathogens (van der Meer-Janssen et al., 2010; Crowley et al., 2013; Vromman and Subtil, 2014; Du et al., 2016). The close contact between host cells and bacteria also facilitates the interchange of phospholipids, as in the case of *B. burgdorferi* and *H. pylori* (Wunder et al., 2006; Crowley et al., 2013; Du et al., 2016; Qaria et al., 2021; Arora et al., 2022; Gwynne et al., 2022).

## 4 Conclusions

Lipids and lipid metabolism are crucial for normal cell functioning, mainly in cells that rely only on β-oxidation as a source of ATP. Subversion of β-oxidation and of functioning of FAT, FABP, and FATP are used by pathogens to acquire free FAs as carbon sources, for energy, and production of other molecules.

Intracellular pathogens evolved mechanisms to exploit cell FA metabolism and membrane lipids to favor their growth and survival. The strategies to accomplish this fate are well reported in the literature, and there are plenty of studies describing the exact mechanism used by those pathogens.

Considering that extracellular bacteria lack the machinery for lipid metabolism and production of cholesterol, the exploitation of lipids from the host’s cell membrane is used as the source of lipids that will compose the bacterial membrane. A close interaction between host cells and bacteria is needed to allow membrane lipids to interchange, and this interaction is mediated by lipid rafts present in both host cell and bacterial membrane.

To date, only a few works have been published addressing the use of lipids and the exploitation of lipid metabolism by extracellular pathogens. Those scarce, but relevant studies, however, shed light on the role of lipids in the interaction between extracellular pathogens and host cells, and point to the need for more experimental studies to uncover the pathways underlying these processes.

## Author contributions

LP wrote the manuscript and conceived the figure. NC and AB revised it critically for intellectual content. All authors contributed to the article and approved the submitted version.
